# A Systematic Review of the Pharmacology, Toxicology and Pharmacokinetics of Matrine

**DOI:** 10.3389/fphar.2020.01067

**Published:** 2020-09-16

**Authors:** Longtai You, Chunjing Yang, Yuanyuan Du, Wenping Wang, Mingyi Sun, Jing Liu, Baorui Ma, Linnuo Pang, Yawen Zeng, Zhiqin Zhang, Xiaoxv Dong, Xingbin Yin, Jian Ni

**Affiliations:** ^1^ School of Chinese Materia Medica, Beijing University of Chinese Medicine, Beijing, China; ^2^ Department of Pharmacy, Beijing Shijitan Hospital Affiliated to Capital University of Medical Sciences, Beijing, China; ^3^ Beijing Research Institute of Chinese Medicine, Beijing University of Chinese Medicine, Beijing, China

**Keywords:** matrine, pharmacology, toxicology, pharmacokinetics, mechanisms

## Abstract

Matrine (MT) is a naturally occurring alkaloid and an bioactive component of Chinese herbs, such as *Sophora flavescens* and Radix *Sophorae tonkinensis*. Emerging evidence suggests that MT possesses anti-cancer, anti-inflammatory, anti-oxidant, antiviral, antimicrobial, anti-fibrotic, anti-allergic, antinociceptive, hepatoprotective, cardioprotective, and neuroprotective properties. These pharmacological properties form the foundation for its application in the treatment of various diseases, such as multiple types of cancers, hepatitis, skin diseases, allergic asthma, diabetic cardiomyopathy, pain, Alzheimer’s disease (AD), Parkinson’s disease (PD), and central nervous system (CNS) inflammation. However, an increasing number of published studies indicate that MT has serious adverse effects, the most obvious being liver toxicity and neurotoxicity, which are major factors limiting its clinical use. Pharmacokinetic studies have shown that MT has low oral bioavailability and short half-life *in vivo*. This review summarizes the latest advances in research on the pharmacology, toxicology, and pharmacokinetics of MT, with a focus on its biological properties and mechanism of action. The review provides insight into the future of research on traditional Chinese medicine.

## Introduction

**Figure 1:**
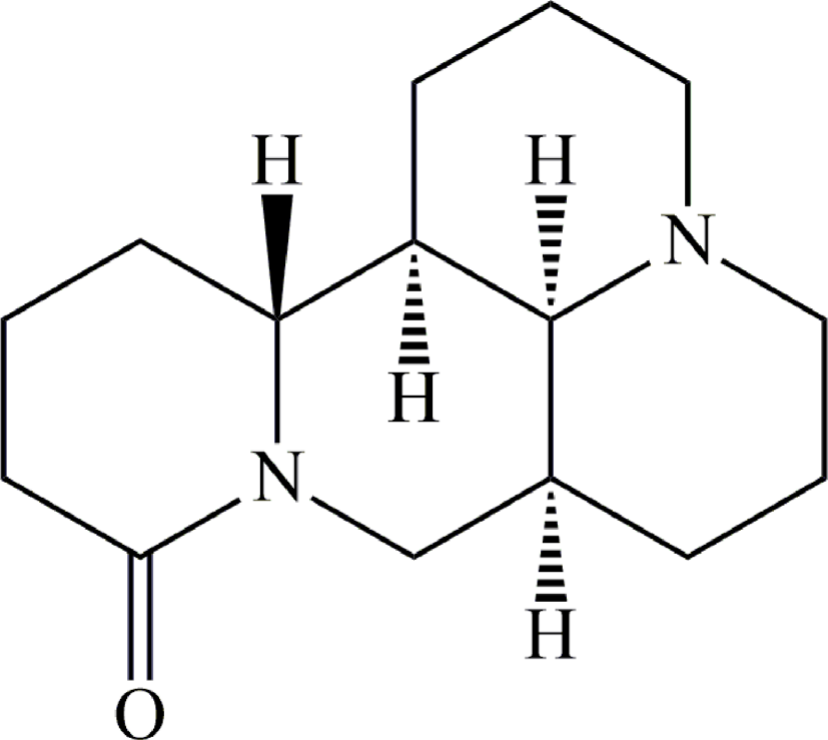
Chemical structure of matrine.

Matrine (MT) (**Figure 1**) is a naturally occurring alkaloid and a bioactive component of Chinese herbs, including *Sophora flavescens* and Radix *Sophorae tonkinensis* (Gu et al., 2019; You et al., 2019). These herbs have a long medicinal history in China and many Eastern Asian countries. In recent years, *in vitro* and *in vivo* studies have demonstrated that MT exhibits linear pharmacokinetics between 5 and 2000 ng/mL (Zhang X. L. et al., 2009), and possesses a wide range of pharmacological eﬀects, including anti-cancer, anti-inflammatory, anti-bacterial, anti-parasitic, anti-virus, anti-fibrotic and, sedative properties (**Table 1**). These pharmacological eﬀects have been exploited for the treatment of hepatitis (Gong et al., 2015); cardiac diseases (Liu et al., 2017b); skin diseases (Liu J. Y. et al., 2007) and many cancers, such as hepatocellular carcinoma (Wang L. et al., 2013); gastric cancer (Luo et al., 2007); breast cancer (Zhou B. G. et al., 2017), and pancreatic cancer (Huang and Xin 2017). Previous reviews reported the chemical composition and anticancer properties of MT (Huang and Xu 2016). However, to date, there are no published comprehensive and systematic reviews on MT. In this review, studies on pharmacology, toxicity, and pharmacokinetics of MT are presented so as to provide comprehensive and updated information on research on MT in the past few decades, and to investigate the therapeutic potential and safety of its components in clinical application.

**Table 1:** Pharmacology of matrine.

Pharmacological effect	Cell lines/model	Activity/mechanism(s) of action		Application	Reference
Anticancer activity	HT29 and DLD1 cells	Triggers mitochondrial apoptotic pathway, Inhibition of p38 signaling pathway		*In vitro* and *vivo*	Chang et al., 2013; Ren et al. (2014)
	K562 cells or primary chronic myeloid leukemia cells	Inhibits IL-6 expression and JAK2/STAT3 pathway		*In vivo*	Ma et al., 2015
	HepG2 and Bel_7402_ cells	Caspase-dependent and caspase-independent apoptotic pathways; inhibition of PI3K/AKT/mTOR pathway; up-regulates expression of Beclin 1		*In vitro*	Zhang et al., 2010; Wang et al., 2013; Zhou et al., 2014;
	SO-RB50 cells	Induces mitochondrial-mediated apoptotic pathway		*In vitro*	Shao et al., 2014
	BxPC-3 and PANC-1 cells	Suppresses the expression of PCNA and a caspase-dependent pathway		*In vitro*	Liu et al. (2010)
	MCF-7 cells	Inhibits the Bcl-2 protein associated with AKT signaling; miR-21/PTEN/AKT Pathway		*In vitro*	Li et al. (2015); Li L. Q. et al. (2012)
	LoVo cells	AKT Pathway		*Invitro*	Zhang et al., 2014
	MKN45 cells	Increases XIAP expression; modulates of NF-κB, p-ERK and CIAP		*In vitro*	Luo et al., 2012
	A549 cells	Down-regulation of VEGF-A and the ratios of Bcl-2/Bax proteins		*In vitro*	Zhang Y. et al. (2009)
	NPC-039 and CNE-2Z cells	Inhibits the expression of NF-κB, MMP-2 and MMP-9		*In vitro*	Sun et al., 2015
	MDA-MB-231 cells	Reduces the activation of MMP-9/MMP-2, phosphorylated AKT, p-65, VEGFR1 and EGF		*In vitro*	Yu et al., 2009
	T24 cells	PI3K/AKT pathway		*In vitro*	Yang et al., 2017
	BCG823 cells	Down-regulates the phosphorylation of VASP		*In vitro*	Zhang J. et al. (2013)
	Y79, WERI-RB1, and SO-RB50 cells	Increases the expression of p21 and p27		*In vitro*	Zhao et al., 2012
	M21 cells	Inhibits the PI3K/Akt pathway; activates p21 expression		*In vitro*	Jin et al., 2013
	SGC-7901 cells	Blocks cell cycle arrest in S phase; inactivation of PI3K/Akt/uPA Pathway.		*In vitro*	Song et al., 2013; Peng et al. (2016)
	Resistant MCF-7/ADR cell line	Decreases the expression of P-gp, MRP1, p-AKT and Bcl-2		*In vitro*	Zhou B. G. et al., 2017
	Resistant K562/DOX cells	Reduces the expression of P-gp		*In vitro*	Chai et al., 2010
	Resistant NCI-H520/TAX25 cells	Reduces the protein and mRNA expression of survivin, Oct-4, and Sox-2		*In vitro*	Luo et al., 2013
	HL-60, THP-1 and C1498 cells	Akt/mTOR signaling inhibition-mediated apoptosis and autophagy pathway; decreases the expression of SQSTM1/p62		*In vitro*	Wu J. et al., 2017
	SKOV3 cells	Decrease the expression of survivin and activate caspase-dependent apoptosis		*In vitro*	Guo et al., 2010
	DLD-1	Induces micropinocytosis and down-regulates ATP level		*In vitro*	Zhang B. et al. (2018)
Anti-inflammatory Activity	NR8383 cells and LPS-induced acute lung injury in mice	Inhibits the expression levels of TNF-α, IL-6 and HMGB1		*In vitro* and *vivo*	Zhang B. et al. (2011)
	Type II collagen-induced arthritis rat	Inhibits the NF-κB pathway and the increases of IL-6, IL-8, IL-1β, IL-17A, TNF-α		*In vivo*	Pu et al., 2016
	Endotoxin-induced acute liver injury after hepatic ischemia/reperfusion in rat	Inhibits the increase of MIP-2, TNF-α and ICAM-1		*In vivo*	Zhang F. et al. (2011)
	Asthmatic mice and TNF-α-stimulated epithelial cells	Suppresses the NF-κB signaling pathway and the expression of SOCS3, (IL)-4 and IL-13		*In vitro* and *vivo*	Sun et al., 2016
	Ovariectomized mice, RAW264.7 and bone marrow monocytes cells	Inhibits RANKL-induced activation of MAPK/AKT/NF-κB pathways		*In vitro* and *vivo*	Chen et al., 2017
	Lps-stimulated A549 cells and mice	Inhibits the activation of NF-κB and MAPK pathways		*In vitro* and *vivo*	Liou et al. (2016)
	Rat intestine microvascular endothelial cells	Inhibits sICAM-1 expression		*In vitro*	Suo et al., 2009
	Human aortic smooth muscle cells	MAPK/NF κB pathway activation		*In vitro*	Liu et al., 2016
	Human chondrocytes	Suppresses expression of MMP-3 and MMP-13; the inhibition of NF-κB and MAPK pathways		*In vitro*	Lu et al., 2015
	Human dermal fibroblasts	Suppresses AP-1 pathway and MMP-1 expression		*In vitro*	Jung et al., 2008
	LPS-stimulated A549 cells	Inhibits COX-2 expression; regulates MAPK/NF-κB activation		*In vitro*	Liou. et al., 2016
	LPS-stimulated mice and Caco-2 cells	Reduces the production of IL-1β, IL-17 and MDA; increases the expression of CCR7		*In vitro*	Wu G. J. et al., 2017
	Injured mouse liver	Inhibits hepatic infiltration of Gr1hi monocytes and MCP-1 activity		*In vitro* and *vivo*	Shi et al., 2013
	Interleukin-10-deficient mice	Reduces the mRNA expression of IL-17 and IFN-γ; down-regulates the proportion of CD4+ T cells of mesenteric lymph node cells		*In vivo*	Wu et al., 2016
	LTA induces endometritis in mice	Inhibits the TLR2-mediated NF-κB pathway		*In vivo*	Jiang K. et al. (2019)
	ox-LDL-induced inflammatory injury of macrophages	Inhibits MKKs/p38 MAPK pathway		*In vivo*	Zhou et al., 2019
	TNBS-induced intestinal inflammation in mice	Improves gut microbiota communities, down-regulates the expression of pro-inflammatory cytokines		*In vivo*	Li P. et al. (2019)
Antioxidant activity	HAEC cells	Suppresses ROS-mediated NRLP3 inflammasome activation		*In vitro*	Zhang et al. (2018b)
	Focal cerebral I/R injury mice	Up-regulates the expression of Bcl-2, SOD, GSH-Px and T-AOC		*In vivo*	Zhao et al., 2015
	Aortic Endothelial Cells	Activates the p38 MAPK/Nrf2/ARE antioxidant signaling pathway		*In vivo* and *vitro*	Liu et al. (2017a)
	High-fructose diet-induced steatohepatitis mice	Activates Nrf2 pathway		*In vivo*	Zhang H. F. et al. (2013)
Antiviral activity	Marc-145 cells	Inactivates PRRSV; blocks the expression of N protein		*In vitro*	Zhao et al., 2013; Sun et al., 2014;
	PK-15 cells	Inhibition of DNA replication of PCV2)		*In vitro*	Sun et al., 2015;
	Porcine kidney cells	Interferes PCV2 Rep protein expression and/or p38/MAPK and JNK/SAPK pathways		*In vitro*	Cheung A. K. (2003); Wei et al. (2009)
	Porcine alveolar macrophages	Inhibits the activation of TLR3, 4/NF-κB/TNF-α pathway		*In vitro*	Na et al., 2016
	HepG2 2.2.15 cell	Inhibits HBV-DNA replication, HBeAg and HBsAg		*In vitro*	Ma et al., 2013
	Rat primary cardiomyocytes	Activiates PI3K/AKT pathway and the expression of Bser-743 protein		*In vitro*	Sun et al., 2010
	Rhabdomyosarcoma cells	Disrupts the replication of enterovirus 71		*In vitro*	Yang et al., 2012
Antimicrobial activity	Gram-negative bacteria	Decreases the expression level of AcrA		*In vitro*	Pourahmad and Mohammadi, 2018
	Staphylococcus epidermidis	Modulates sigB, agr and fbe virulence genes		*In vitro*	Zhu (2014)
Anti-nociceptive Activity	Kunming mice	Regulates the kappa-opioid receptor and the μ-opioid receptors		*In vivo*	Qian et al., 2012
	SD mice	Inhibits the expression of TNF-α in dorsal root ganglion and Ras/Raf/ERK1/2 pathway		*In vivo*	Tao et al., 2013; Gong et al. (2016)
Hepatoprotective activity	High-fructose diet-induced rat	Activates Nrf2 pathway		*In vivo*	Zhang H. F. et al., 2013
	High-fat-fed mice	Increases the mRNA and protein level of HSP72 in liver tissues		*In vivo*	Zeng et al., 2015
	HIR rat model	Suppresses myeloperoxidase, NF-κB activity and macrophage-inflammatory protein-2 expression		*In vivo*	Zhang F. et al., 2011
	Rat Kupffer cells and immunological liver injury model in BALB/c Mice	Inhibits the expression of inflammatory factors		*In vivo* and *vitro*	Lin et al. (1997); Yu S. X. et al., 2009
	HSC-T6 cells	Inhibits cell proliferation and reduces collagen I and HA levels.		*In vivo*	Zhao et al., 2012
	Patients with chronic hepatitis B	Restores liver function and transforms serum from hepatitis Be antigen to HBe antibody and serum HBV DNA from positive to negative		*In vivo*	Long et al. (2004)
	CCl_4_-induced acute liver injury rat	Prevents Gr1hi monocyte infiltration f and inhibits MCP-1 production		*In vivo*	Shi et al., 2013
	CCl_4_-induced rat liver fibrosis model	MT salvianolic acid B salt decreases the expressions of HA, LN, AST, ALT, Hyp, TGF-β1 and α-SMA.		*In vivo*	Gao et al., 2012
Cardioprotective activity	Rat cardiac microvascular endothelial cells	Activates the JAK2/STAT3 signaling pathway		*In vivo*	Zhao et al., 2018
	Rat cardiomyocytes	Regulates HSP70 Expression by the Activation of JAK2/STAT3 pathway		*In vivo*	Guo S. et al., 2018
	Isoproterenol-induced acute myocardial ischemia in rat	Regulates the expression of ADMA and eNOS; inhibits the release of cTn-I and RhoA/ROCK1 pathway		*In vivo*	Li X. et al., 2010; Li X. et al., 2012; Zhou et al., 2016
	DCM rat	Inhibition of ROS/TLR-4, ATF6 or TGF-β1/Smad pathways	*In vivo*		Liu et al., 2015; Zhou J. et al., 2016; Liu et al., 2017b
	Heart failure model in rats.	Blocks myocardial apoptosis and β3−adrenoreceptor/endothelial nitric oxide synthase pathway	*In vivo*		Yu et al., 2014
Neuroprotective activity	Aβ1-42-induced AD rat	Reverses the imbalance of Th17/Treg cytokines	*In vivo*		Zhang. et al., 2015
	AD transgenic mice	Blocks the Aβ/RAGE signaling pathway	*In vivo*		Cui et al., 2016
	MPTP -induced PD rat	Blocks the overexpression of Nrf2	*In vivo*		Meng et al., 2017
	Autoimmune encephalomyelitis in mice	Enhances the local production of NT3 in CNS cells; activates PI3K/Akt/mTOR pathway; blocks NogoA-induced neural inhibitory signaling pathway	*In vivo*		Kan et al. (2016); Liu S. Q. et al., 2017; Zhang M. L. et al., 2017
	LPS-stimulated BV-2 cells	Suppresses the expression of HSP60 and TLR-4	*In vitro*		Zhang R. et al., 2017
	LPS-induced AD mice	Inhibits the protein expression of GP91phox and P47phox	*In vivo*		Li et al., 2018
	Burned rat	Improves anxiety and depression symptoms by inhibiting JNK-mediated apoptosis/inflammatory signaling, oxidative stress, and increases of BDNF/VEGF in hippocampus.	*In vivo*		Khan et al., 2020
	Chronic unpredictable mild stress mouse model of depression	Activation of PI3K/Akt/mTOR signaling pathway in hippocampus	*In vivo*		Wu et al., 2018
Anti-allergic activity	OVA-sensitized mice	Inhibits Th2 cytokines or IL-4/IL-13/STAT-6 pathway	*In vivo*		Fu et al., 2014
	BEAS-2B cells	Inhibits the expression of ICAM-1, pro-inflammatory cytokines and eosinophil chemokines	*In vitro*		Huang et al. (2014)

## Pharmacology

### Anticancer Effects of MT

#### Induction of Apoptosis

Apoptosis is a physiological process of autonomous, programmed cell death that is used to remove malignant cells such as cancer cells without causing damage to normal cells or surrounding tissues. Apoptosis induced by MT has been reported in diverse human cancer cells. Treatment of human hepatocellular carcinoma HepG2 and Bel_7402_ cells with MT at doses of 0.2–3.2 mg/mL for 24, 48, and 72 h caused a dose- and time-dependent apoptosis (Wang L. et al., 2013). Moreover, MT inhibited cell growth and induced apoptosis in HT29 cells by regulating the expressions of apoptosis-related genes. Incubation with MT enhanced apoptosis in HT29 cells *via* the release of cytochrome c in cytoplasm and the up-regulation of apoptotic-related genes including caspase-3, caspase-9, and Bax/Bcl-2 ratio (Chang et al., 2013). In another study, MT treatment significantly inhibited cell proliferation and induced apoptosis in K562 cells (primary chronic myeloid leukemia cells) through regulation of genes related to the JAK2/STAT3 signaling pathway (Ma et al., 2015). In that study, inhibition of the expression of IL-6 expression subsequently decreased the protein expressions of downstream JAK2 and STAT3 (Ma et al., 2015).

MT effectively induced programmed cell death in HepG2 cells through caspase-dependent and caspase-independent apoptotic pathways involving the loss of mitochondrial membrane potential, reactive oxygen species (ROS) generation, Bid-mediated AIF nuclear translocation, and cytochrome c release from the mitochondria. Moreover, MT treatment up-regulated the expression of apoptosis-related proteins Fas/Fas-L and cleaved caspase-3, while down-regulating the expressions of procaspase-3, procaspase-8, and procaspase-9 (Zhou et al., 2014). It has been reported that MT inhibited the proliferation of SO-RB50 cells and initiated apoptosis by activating the caspase family of proteins, resulting in disruption of mitochondrial permeability transition pores (Shao et al., 2014). Moreover, MT treatment triggered apoptosis in human pancreatic cancer cells (BxPC-3 and PANC-1) *via* decreases in the expressions of PCNA and a caspase-dependent pathway (Liu et al., 2010).

The AKT signaling pathway is an important cell signaling pathway upstream of many genes that regulate cell survival, proliferation, angiogenesis, and metabolism (Manning and Cantley, 2007). Previous studies have shown that MT exerts anti-cancer effects in human breast cancer cells (MCF-7) by inhibiting Bcl-2 protein associated with the AKT signaling pathway (Li et al., 2015). A similar study indicated that MT inhibited the growth of breast cancer MCF-7 cells by modulating the miR-21/PTEN/AKT pathway (Li L. Q. et al., 2012). Interestingly, MT also inhibited human colon cancer LoVo cell proliferation through inactivation of the AKT pathway (Zhang et al., 2014). Furthermore, Guo et al., demonstrated that MT effectively inhibited the proliferation of human ovarian cancer cells (SKOV3), and its potential mechanism might be to decrease the expression of survivin and activate caspase-dependent apoptosis (Guo et al., 2010).

The X- chromosome-linked inhibitor of apoptosis protein (XIAP), an anti-apoptotic factor from the IAP family, suppresses caspase activity (Wrzesień et al., 2004). Recent studies have demonstrated that MT treatment induced apoptosis in human gastric cancer cells (MKN45) by increasing the XIAP protein expression level, modulating NF-κB, phosphorylated extracellular signal-regulated kinase (p-ERK) and CIAP (Luo et al., 2012). Interestingly, Zhang B. et al. (2018) reported that MT could induce macropinocytosis in human colon adenocarcinoma cells (DLD-1) and down-regulate ATP level in the cells, leading to non-apoptotic cell death. This provides a new strategy for the development of MT as an anticancer drug.

#### Anti-Metastatic Effect of MT

Tumor metastasis is a complex process that entails decreased tumor cell adhesion and degradation and remodeling of the extracellular matrix (ECM) (Hsieh et al., 2014). Studies have shown that MT significantly inhibited A549 cell growth and migration through the down-regulation of vascular endothelial growth factor A (VEGF-A) and changes in the ratio of Bcl-2/Bax proteins (Zhang Y. et al., 2009). In another study, MT at doses of 12.5, 25, 50, 100, and 200 µg/mL, inhibited the migration and invasion of nasopharyngeal carcinoma cells (NPC-039 and CNE-2Z) through suppression of the expression of NF-κB and down-regulation of downstream matrix metalloproteinase-2 and metalloproteinase-9 (MMP-2 and MMP-9) proteins (Sun and Min, 2015). It has been demonstrated that MT effectively inhibited the invasion of highly-metastatic human breast cancer MDA-MB-231 cell *in vitro* by reducing the activation of MMP-9/MMP-2, enhancing the phosphorylation of AKT, and decreasing the activities of p-65, VEGFR1, and epidermal growth factor (EGF) (Yu P., et al., 2009). In addition, Yang et al. reported that MT dose-dependently inhibited the growth and invasion of bladder cancer T24 cells *via* the PI3K/AKT pathway, and regulation of invasion-related genes (Yang Y. et al., 2017).

It has been demonstrated that MT significantly inhibited cell proliferation and invasion in colorectal cancer cells (HT29 and DLD1) *via* reduction in the activation of the p38 signaling pathway (Ren, 2014). Another study demonstrated that MT significantly down-regulated the expression and phosphorylation of vasodilator-stimulated phosphoprotein (VASP), which is up-regulated in gastric cancer cells (BCG823), thereby suppressing tumor cell migration and adhesion (Zhang J. et al., 2013).

#### Effect of MT on Cell Cycle

Impairment of cell cycle regulation is an important process in malignant transformation. Cyclins, cyclin-dependent kinases, and their inhibitors are involved in the regulation of cell cycle progression (Shi et al., 2003). Previous studies showed that MT dose-dependently inhibited the proliferation of human retinoblastoma cells (Y79, WERI-RB1, and SO-RB50), and induced cell cycle arrest at the G0/G1 phase in a time-dependent manner. The intracellular mechanisms involved are related to increased levels of the CDK inhibitors p21 and p27, and the decreased levels of the cyclin D1 protein (Zhao et al., 2012). Melanoma M21 cells also showed similar effects (Jin et al., 2013). Moreover, studies have revealed that MT caused cell cycle arrest in S phase in gastric cancer SGC-7901 cells (Song et al., 2013; Peng et al., 2016). In addition, MT has been reported to up-regulate the expressions of p53 and p21, and down-regulate the expressions of CDK2, CDK4, cyclin D1, cyclin E, and phosphorylated Rb, leading to the arrest of vascular smooth muscle cells (VSMCs) at the G0/G1 phase (Zhu et al., 2010).

#### Reversion of Multidrug Resistance

Chemotherapy is the major treatment for various cancers. However, excessive expression of multi-drug resistance (MDR) in tumor cells has seriously affected the success of chemotherapy. The regulation of MDR gene expression is a complex process that is primarily associated with increases in a variety of adenosine triphosphate (ATP)-binding cassette transporters, including P-glycoprotein (P-gp), and multidrug resistance-related protein (MRP) (Hien et al., 2010; Kanagasabai et al., 2011). Previous studies have demonstrated that MT (0-2.5 mg/mL) increased the intracellular accumulation of adriamycin (ADR) and triggered its apoptotic effects in the resistant MCF-7/ADR cell line through decreased expressions of P-gp, MRP1, p-AKT, and Bcl-2 (Zhou B. G. et al., 2017). Another study also found that MT up-regulated the intracellular accumulation of doxorubicin (DOX), and induced apoptosis of K562/DOX cells through a reduction in the expression of P-gp (Chai et al., 2010). Moreover, recent studies have shown that excessive expression of survivin is closely associated with MDR (Wang Q. P. et al., 2013). Decreased mRNA and protein expressions of survivin, Oct-4, and Sox-2 may be the molecular mechanism involved in the MT-mediated reversal of paclitaxel (TAX) resistance in NCI-H520/TAX25 cells (Luo et al., 2013).

#### Induction of Autophagy

Autophagy is an important process of cell death through which cells degrade and circulate their own components, including rapid caspase-independent self-digestion, nuclear condensation, organelle swelling, and lysosomal degradation (Yang et al., 2011; Kondratskyi et al., 2017). In addition, apoptosis and autophagy are regulated by subtle crosstalk, and their signaling pathways are interrelated in various diseases (Wu et al., 2014). Previous studies have reported that MT inhibited the growth of HepG2 cells *via* induction of apoptosis and autophagy. Following MT treatment, HepG2 hepatoma cells showed obvious morphological changes, including the occurrence of a large number of autophagic vacuoles (AVs) of different sizes, and the up-regulated expression of Beclin 1, which was the first identified autophagy-inducing mammalian gene (Zhang et al., 2010). Moreover, Wang et al. showed that the inhibition of autophagy promoted apoptosis of human hepatoma cells induced by MT (Wang L. et al., 2013). In acute myeloid leukemia cell lines (HL-60, THP-1 and C1498), treatment with MT at doses of 0.25-3 g/L for 12-48 h resulted in cytotoxicity *via* induction of Akt/mTOR signaling inhibition-mediated apoptosis and autophagy, which is involved in increased expression of LC3-II and decreased SQSTM1/p62 ratio (Wu J. et al., 2017). Therefore, MT seems to affect both autophagy and apoptosis through crosstalk.

These findings indicate that MT inhibits the growth of various cancer cells and regulates the expressions of genes and proteins associated with apoptosis, autophagy, cell invasion, metastasis, and cell cycle arrest.

### Anti-Inflammatory Effect of MT

The anti-inflammatory effect of MT been well confirmed. This effect is exerted *via* regulation of the expressions of inflammatory cytokines and chemokines such as tumor necrosis factor-α (TNF-α), interleukin (IL)-1, IL-2, IL-4, IL-5, and IL-10; pro-inflammatory transcription factors, i.e., nuclear factor kappa-B (NF-κB), and inflammatory mediators, i.e., nitric oxide and matrix metalloproteinases (NO and MMPs). MT has been used for the treatment of rheumatoid arthritis, hepatitis, atopic dermatitis, endometritis, and enteritis (Zhang B. et al., 2011; Zhang L. et al., 2015; Jiang K. et al., 2019).

The potential mechanisms of the anti-inflammatory effects of MT may involve the following:

1. Down-regulation of the expressions of inflammatory cytokines by inhibiting NF-κB.MT exerts its anti-cancer activity, partly at least, through the inhibition of NF-κB. It also plays an extensive role in regulating the inflammatory response through the NF-κB signaling pathway. In a type II collagen-induced arthritis rat model, MT exerted its anti-arthritis effects *via* down-regulation of pro-inflammatory cytokines and proteins (IL-6, IL-8, IL-1β, IL-17A, and TNF-α) and down-regulation of NF-κB (Pu et al., 2016). In addition, MT inhibited lipopolysaccharide (LPS)-induced increases in macrophage inflammatory protein-2 (MIP-2), TNF-α, soluble intercellular adhesion molecule-1 (ICAM-1), and NO through inhibition of the activity of NF-κB (Zhang F. et al., 2011). It has been reported that MT inhibited ovalbumin (OVA)-induced airway hyperresponsiveness (AHR) in mice by decreasing the production of IL-4 and IL-13, and increasing the expression of interferon (IFN)-γ. Furthermore, the study showed that MT suppressed the expression levels of SOCS3 in asthmatic mice and TNF-α-stimulated epithelial cells (BEAS-2B and MLE-12) by inhibiting the NF-κB signaling pathway (Sun et al., 2016). Estrogen withdrawal results in up-regulation of pro-inflammatory cytokines, thereby inducing overactivation of osteoclasts inflammation. Recent studies have shown that MT significantly prevented ovariectomy-induced osteoporosis and osteoclastogenesis *in vivo*, while reducing serum levels of IL-6, TNF-α, and tartrate-resistant acid phosphatase 5B (TRA_CP_5B). Moreover, MT significantly inhibited osteoclast differentiation of RAW264.7 and bone marrow monocytes cells though the inhibition of NF-κB ligand (RANKL)-induced activation of MAPK/AKT/NF-κB pathways (Chen et al., 2017). Another study showed that MT has a protective effect on Staphylococcus aureus lipoic acid (LTA) induced endometritis by inhibiting the TLR2-mediated NF-κB pathway (Jiang K. et al., 2019).
2. Decreases in the expressions of endothelial cell adhesion molecules (ECAMs). Decreased expressions of ECAMs have been shown to reduce the vascular complications induced by inflammation (Hu et al., 2013). The transcription and expression of intercellular adhesion molecule-1 (ICAM-1) could be decreased *via* treatment with MT in LPS-stimulated human lung epithelial A549 cells *via* inhibition of the activation of NF-κB and MAPK pathways (Liou et al., 2016). Moreover, MT decreased LPS-induced increases in IL-6, IL-8, and soluble intercellular adhesion molecule-1 (sICAM-1) in rat intestine microvascular endothelial cells (RIMECs) (Suo et al., 2009). In addition, it has been found that MT treatment inhibited TNF-α-induced up-regulation of vascular cell adhesion molecule-1 (VCAM−1) and ICAM−1 in human aortic smooth muscle cells through the activation of MAPK/NF-κB pathway (Liu et al., 2016).
3. Reduction of the expressions of MMPs. Matrix metalloproteinases (MMPs) are involved in tumorigenesis, tumor invasion and inflammation-related diseases, e.g., osteoarthritis. It is known that MMP-3 and MMP-13 are responsible for the degradation of extracellular matrix and are associated with cartilage degradation. Studies demonstrated that MT effectively suppressed IL-1β-induced expressions of MMP-3 and MMP-13 in human articular cartilage chondrocytes *via* inhibition of the activation of the NF-κB and MAPK signaling pathways (Lu et al., 2015). Moreover, MT significantly inhibited the mRNA and protein expressions of MMP-1 induced by phorbol myristate acetate (PMA) through suppression of the activation of the AP-1 signaling pathway (Jung et al., 2008).
4. Suppression of the production of cyclooxygenase-2 (COX-2). COX-2 induces inflammation and enhances capillary permeability through multiple stimuli, including injury, tumorigenesis, migration, and control of prostaglandin production (Flower, 2003). Studies have shown that MT possesses significant COX-1 and COX-2 inhibitory activity (Ao et al., 2009). Moreover, MT treatment alleviated the LPS-induced inflammatory response and inhibited the gene expression of COX-2 in LPS-stimulated A549 cells through regulation of the activation of MAPK/NF-κB (Liou et al., 2016).
5. Other mechanisms. Besides the above-mentioned mechanisms, MT also moderates inflammation in other ways. In mice and Caco-2 cell models, MT alleviated LPS-induced inflammation and oxidative stress through reduction in the production of IL-1β, IL-17, and malondialdehyde (MDA), while increasing the expressions of chemokine receptor 7 (CCR7) (Wu G. J. et al., 2017). Furthermore, MT exhibited anti-fibrotic eﬀects, as demonstrated *via* suppression of hepatic infiltration of Gr1hi monocytes and inhibition of the activity of monocyte chemoattractant protein-1 (MCP-1) (Shi et al., 2013). Moreover, MT effectively reduced the mRNA expressions and cytokines (IL-17 and IFN-γ) in interleukin-10-deficient mice, and down-regulated the proportion of CD4+ T cells of mesenteric lymph node cells (Wu et al., 2016). It was also found that MT inhibited the oxidized low-density lipoprotein (ox-LDL)-induced inflammatory injury of macrophages by inhibiting the MAP kinase kinases (MKKs)/p38 MAPK signaling pathway (Zhou et al., 2019). It is worth noting that MT effectively alleviated colonic injury and intestinal inflammation by improving gut microbiota communities (i.e., Bacilli and Mollicutes), down-regulating the expression of pro-inflammatory cytokines (IL-1 and TNF-α), and increasing serum immunoglobulin G (IgG) (Li P. et al., 2019). This provides a new direction and reference for the development of MT targeting intestinal flora.

### Antioxidant Property of MT

Excessive generation of ROS is involved in various pathophysiological processes such as cancer, aging, chronic inflammation, neurodegenerative diseases, and degenerative rheumatic diseases. It has been reported that MT suppressed advanced glycation end products (AGEs)-induced ROS production in human aortic endothelial cells (HAEC) (Zhang et al., 2018b). In the middle cerebral artery occlusion (MCAO)-induced focal cerebral ischemia-reperfusion (I/R) injury model, MT pretreatment for 7 consecutive days effectively decreased Bax and, caspase-3 expressions, and reduced MDA levels, while it up-regulated Bcl-2, superoxide dismutase (SOD), glutathione peroxidase (GSH-Px), catalase (CAT), and total antioxidant capacity (T-AOC), thereby attenuating MCAO-induced cerebral I/R injury (Zhao et al., 2015). In addition, MT markedly increased the expression levels of antioxidant enzymes such as NADPH quinone oxidoreductase-1(NQO-1) and heme oxygenase-1(HO-1), and decreased AGEs-induced ROS production through activation of the p38 mitogen-activated protein kinase/nuclear factor E2-related factor-2/antioxidant response elements (MAPK/Nrf2/ARE) antioxidant signaling pathway (Liu et al., 2017a). In a D-galactose- (D-gal-) induced aging mouse model, Sun et al. found that MT inhibited oxidative stress damage in the liver, plasma, and brain of mice by increasing T-AOC, T-SOD, CAT, and decreasing MDA levels. Meanwhile, MT suppressed the activation of p19/p21 and p16 pathways in the liver and hippocampus of D-gal-induced mice. These results suggest that MT plays an anti-aging role by inhibiting oxidative stress and cell senescence (Sun et al., 2018).

### Antiviral Property of MT

Porcine reproductive and respiratory syndrome (PRRS) is a devastating swine disease caused by a genetically diverse RNA virus that directly affects the economics of the swine industry. Previous studies have shown that MT suppressed porcine reproductive and respiratory syndrome virus (PRRSV) infection in Marc-145 cells through direct inactivation of PRRSV, and interference with its cellular replication (Zhao et al., 2013). Subsequent studies found that MT significantly blocked the expression of N protein in Marc-145 cells and inhibited PRRSV-induced apoptosis through the suppression of caspase-3 activation (Sun et al., 2014). Moreover, Sun et al. reported that the exposure of porcine kidney cell line (PK-15) to MT resulted in a dose-dependent inhibition of DNA replication of porcine circoviruses 2 (PCV2) (Sun et al., 2015). The anti-PCV2 mechanism of MT may be related to interference with PCV2 Rep protein expression and/or interference with host cell p38/MAPK and JNK/SAPK signaling pathways (Cheung, 2003; Wei et al., 2009). Another study showed that MT exerted anti-PRRSV/PCV2 co-infection activity *in vitro via* the inhibition of the activation of TLR3 and 4/NF-κB/TNF-α pathways (Na et al., 2016). These results indicate that MT may be considered a major potential therapeutic drug for PRRS.

Previous studies found that MT inhibited wild-type and entecavir-resistant hepatitis B virus (HBV) *in vitro*, and effectively inhibited HBV replication *in vivo* (Liu et al., 2018). Moreover, MT interfered with infection of coxsackievirus group B 3 (CVB3) (Liu et al., 2003). Similarly, it has been found that MT inhibited CVB3 virus-induced neonatal rat cardiomyopathy, reduced the rate of apoptosis, and up-regulated the expression of phosphorylated protein kinase Bser-743 protein. The results showed that MT repaired CVB3 virus-induced cell damage through up-regulation of the phosphatidylinositol 3-kinase/protein kinase B (PI3K/AKT) signaling pathway (Sun et al., 2010). The *in vitro* inhibitory effect of MT in a model of human laryngeal carcinoma epithelial cells (Hep-2 cells) infected with respiratory syncytial virus, has been reported (Ma et al., 2002). Furthermore, MT exerted antiviral effects by inhibiting the viral RNA copy number on rhabdomyosarcoma cells, and disrupting the replication of enterovirus 71 in the mouse model (Yang et al., 2012). These results indicate that MT has a broad-spectrum antiviral potential that merits further investigation.

### Antimicrobial Effect of MT

MT showed a remarkable bacteriostatic effect on *Candida albicans* SC5314. The bioactive minimum inhibitory concentration (MIC_80_) and effective concentration (EC50) values of MT were 1 and 2 mg/mL, respectively. In addition, MT reversed fluconazole -resistance of *Candida albicans* 215, either in the biofilm phenotype or in the free-floating form. The MIC_80_ and EC_50_ of MT were 3 and 6 mg/mL, respectively. These results indicated that MT suppressed *Candida*-related infections by controlling yeast-to-hypha conversion (Shao et al., 2015). Similarly, it has been reported that MT exerted strong inhibitory effects on *E. coli, Bacillus subtilis*, and *S. aureus* with MICs of 12.5, 12.5, and 25 μg/mL, respectively (Liu et al., 2011). Zhao et al. investigated the antibacterial effects of MT on drug-resistant *E. coli* and methicillin-resistant *Staphylococcus aureus* (MRSA) isolated from the uterus of dairy cows. The results showed that MT significantly inhibited the growth of drug-resistant *E. coli* (MIC = 12.5 mg/mL) and MRSA strains (MIC = 25 mg/mL) (Zhao and Yu, 2017). AcrAB-TolC is a three-component pump that includes cytoplasmic membrane proteins (AcrB), periplasmic proteins (AcrA), and outer membrane protein channels (TolC); over-activation of this pump resulted in multidrug-resistant (MDR) strains.

MT modulated *sigB*, *agr*, and *fbe* virulence genes of *Staphylococcus epidermidis* isolated from milk samples of clinical mastitis, down-regulated *sigB* and *fbe* genes, up-regulated the *agr* gene, and promoted the *atlE* expression of gene and synthesis of phenol-soluble peptide (PSMs), resulting in rapid shedding of bacteria from the biofilm (Zhu, 2014). In recent years, it has been reported that the Panton-Valentine leukocidin (PVL) toxin of *Staphylococcus aureus* may be related to the pathogenic mechanism of bovine mastitis. Jia et al. found that MT inhibited PVL-induced apoptosis of bovine mammary epithelial cells (BMEC) by down-regulating the expression of cleaved caspase-3, cleaved caspase-8, and cleaved caspase-9 proteins (Jia et al., 2020). Furthermore, a subinhibitory concentration of MT inhibited the *Staphylococcus aureus* secretion of α-hemolysin in a none dose-dependent manner, thus reducing the inflammatory damage of α-hemolysin-induced BMEC cells (Feng et al., 2018). These results suggest that MT may be developed into a novel drug for the prevention and treatment of bovine mastitis. Generally speaking, MT may be considered a potential antibacterial drug for further research and development.

### Anti-Nociceptive Activity

Studies have demonstrated that subcutaneous injections of MT at doses of 1-10 mg/kg reduced the number of acetic acid-induced writhing responses induced, with ED_50_ of 4.7 mg/kg (Kamei et al., 1997). Other studies have reported that subcutaneous injections of MT at dose of 10-100 mg/kg dose-dependently inhibited hot water induced tail-flick response in mice, with pick analgesic effect after 30 min (Xiao et al., 1999). These results imply that MT produced anti-nociceptive effects mainly through the kappa-opioid receptor, and partially through the μ-opioid receptors. However, after an intravenous injection of MT at doses of 10, 20, and 40 mg/kg, the percentage inhibition values of acetic acid-induced writhing responses in mice were 15, 25, and 55, respectively, indicating that the anti-nociceptive effect was not significant, but it had obvious anti-inflammatory effects (Qian et al., 2012). Moreover, another study showed that MT reduced neuropathic pain in a dose-dependent manner by inhibiting the expression of tumor necrosis factor-α in dorsal root ganglion (Tao et al., 2013). Studies have shown that MT (15-60 mg/kg, i.p.) had an anti-nociceptive effect on mechanical stimulation and cold stimulation of vincristine induced neuropathic pain in model mice (Dun L. L. et al., 2014). In addition, it has been shown that MT reduced vincristine-induced neuropathic pain in mice through mechanisms associated with down-regulation of expression of spinal cord Ras, phosphorylated c-Raf, p-ERK 1/2, TNF-α, and IL – 6, and up-regulation of the expression of IL-10 (Gong et al., 2016). Overall, MT has potent analgesic effects which should be exploited in drug research and development for the benefit patients suffering from pain.

### Hepatoprotective Effect of MT

It has been demonstrated that MT at doses of 40, 80, and 160 mg/kg/day for 28 days mitigated high-fructose diet (HFD)-induced hepatic steatosis, and reduced the expression of aspartate aminotransferase (AST) and alanine aminotransferase (ALT), while reducing malondialdehyde and increasing reduced glutathione. Moreover, MT significantly promoted Nrf2 translocation to the nucleus, and subsequently increased the protein expressions of the antioxidative enzymes, CAT, SOD, and GSH-Px (Zhang H. F. et al., 2013). Furthermore, Zhang et al. have shown that MT exerted antioxidant capacity not only by reducing MDA levels and increasing liver GSH production, but also *via* stimulating Nrf2 translocation and the expressions of its downstream antioxidant enzymes GSH-Px, SOD, CAT, HO-1, and NQO-1, suggesting that the hepatoprotective effects of MT on the liver may be related to its restoration of liver redox balance (Zhang H. F. et al., 2013).

In a high-fat-fed mouse model, MT at a dose of 100 mg/kg/day for 28 days, reduced glucose intolerance and plasma insulin levels, hepatic triglyceride levels, and obesity, without affecting caloric intake. Moreover, MT increased the mRNA and protein levels of heat shock protein-72 (HSP72) in liver tissues (Zeng et al., 2015). Furthermore, it has been reported that MT (100 mg/kg/day for 4 weeks) effectively reduced glucose intolerance and plasma insulin levels in high-fructose-fed mice by inhibiting endoplasmic reticulum (ER) stress-associated *de novo* lipogenesis (DNL) and increasing HSP72 protein expression in the liver (Mahzari et al., 2018). These results suggest that the hepatoprotective drug MT may be a promising novel anti-type 2 diabetes drug, and the liver is its important target organ.

In another experiment, MT at a dose of 100 mg/kg/day for 7 days, was shown to attenuate endotoxin-induced acute liver injury after hepatic ischemia/reperfusion (HIR) in rats by suppressing myeloperoxidase, nuclear factor κB activity, and macrophage-inflammatory protein-2 expression in a dose-dependent manner (Zhang F. et al., 2011). This indicates that MT might be used for the treatment of HIR-induced liver injury. It has been demonstrated that various doses of MT (50 or 100 mg/kg/day for 5 days; 20, 40, or 80 mg/kg/day for 10 days attenuated LPS-induced liver injury through modulation inflammation (Lin et al., 1997; Yu S. X. et al., 2009). Furthermore, MT at a dose of 10 mg/kg (*i.p.*) exhibited hepatoprotective effect against α-naphthyl isothiocyanate (ANIT)-induced liver damage *via* the inhibition of the ANIT-induced increase in serum ALT, alkaline phosphatase (ALP), total bilirubin (Tbil), and γ-GT (Zhai et al., 2007).

Fibrosis is one of the common causes of chronic organ failure. It is characterized by the accumulation of extracellular matrix and the destruction of normal tissue structures (Wynn, 2007). Recent evidence indicates that MT has been used as an anti-fibrotic agent to treat hepatic disorders. The anti-fibrotic eﬀects of MT (10 or 30 mg/kg/day, given 5 times twice in a week for 3 weeks) has been attributed in part to the inhibition of Gr1^hi^ monocytes to injured liver, and the inhibition of production/activity of monocyte chemo attractant protein-1 (MCP-1) (Shi et al., 2013). Moreover, administration of MT salvianolic acid B salt at a dose of 25, 50, or 100 mg/kg/day for 56 days led to significant amelioration of fibrotic changes and decreased expressions of HA, LN, AST, ALT, hydroxyproline (Hyp), transforming growth factor beta 1 (TGF-β_1_), and alpha-smooth muscle actin (α-SMA). The depletion of reduced glutathione (GSH), and SOD accumulation in liver tissues were inhibited by MT salvianolic acid B salt. These results indicate that treatment with MT salvianolic acid B salt mitigated carbon tetrachloride-induced fibrosis, implying that MT is a potential anti-fibrotic agent (Gao et al., 2012). In addition, MT (100 mg/day for 90 days) was found to be effective in improving the clinical symptoms and signs of patients with chronic hepatitis B, restoring liver function and transforming serum from the hepatitis Be antigen to the HBe antibody and serum HBV DNA from positive to negative. The total effective rate of the MT group was 86.7%, and no serious side effects were observed except for a few patients with mild pain at the intramuscular injection site of MT (Long et al., 2004).

### Cardioprotective Effect of MT

Previous studies showed that the JAK2/STAT3 signaling pathway is involved in the prevention of myocardial ischemia/reperfusion (I/R) injury (Boengler et al., 2008). Indeed, MT activates the JAK2/STAT3 pathway. Studies have shown that MT inhibited hypoxia/reoxygenation (H/R)-induced apoptosis of cardiac microvascular endothelial cells (CMECs) in rats, through increases in the phosphorylation of the JAK2/STAT3 signaling pathway-related proteins (Zhao et al., 2018). A similar study suggested that MT effectively up-regulated the expression of heat shock protein 70 (HSP70) and reduced lactate dehydrogenase (LDH) release, decreased activity of creatine kinase-myocardial band (CK-MB), and mitigated cardiomyocytes apoptosis *via* activation of the JAK2/STAT3 signaling pathway (Guo Q. P. et al., 2018). These data indicate that MT can be used as a potential JAK2/STAT3 signaling pathway activator in studies of its cardioprotective effects. Furthermore, another study confirmed that MT possesses a protective effect on rat heart failure *via* blocking myocardial apoptosis and β3−adrenoreceptor/endothelial nitric oxide synthase pathways (Yu et al., 2014). Moreover, results have shown that MT plays an important role in cardiovascular protection in isoproterenol-induced acute myocardial injury *via* its antioxidant properties. The protective effect of MT on isoproterenol-induced acute myocardial ischemia in rats was associated with the regulation of asymmetric dimethylarginine (ADMA) and endothelial nitric oxide synthase (eNOS) (Li X. et al., 2010; Li X. et al., 2012). Moreover, MT up-regulated the expressions of transforming growth factor-β1 (TGF-β1) and insulin-like growth factor-1 (IGF-1), while inhibiting the release of cardiac troponin (cTn-I) and suppressing activation of inflammatory mediator RhoA/ROCK1, thereby exerting a protective effect on isoproterenol-induced acute myocardial ischemia (Zhou et al., 2016).

Diabetic cardiomyopathy (DCM) is one of the causes of disability or death in diabetic patients, and it is often accompanied by persistent hyperglycemia, metabolic disorders, and cardiac fibrosis (Cai and Kang, 2003; Cai, 2007). Administration of MT (200 mg/kg/day for 10 days, *po*) improved cardiac dysfunction in DCM rats, most likely *via* suppression of the activation of the ROS/TLR-4 signaling pathway and reduction of the expression of myocyte apoptosis-related proteins such as caspase-8 and caspase-3 (Liu et al., 2015). It was reported that MT significantly improved cardiac function and compliance by inhibiting the ATF6 signaling pathway in DCM rats (Liu et al., 2017b). In addition, MT inactivated the TGF-β1/Smad signaling pathway by inhibiting the expression of TGF-β1 and phosphorylation of Smad2/3 in a dose-dependent manner, thereby exerting anti-fibrotic effects in DCM rats (Zhang et al., 2018a).

These results indicate that MT ameliorates the symptoms of diabetic cardiomyopathy through inhibition of the ROS/TLR-4, ATF6, or TGF-β1/Smad signaling pathways. Therefore, MT may be considered a potential therapeutic agent for diabetic cardiomyopathy and its complications.

### Neuroprotective Effect of MT

Deposition of the amyloid-β protein (Aβ) in the tight structure between neurons is closely linked to the pathogenesis of AD, and its accumulation in the hippocampus and neocortex may lead to neuronal death and eventually irreversible cognitive impairment and behavioral changes (Dong et al., 2018). Studies have shown that MT, at doses of 100 and 200 mg/kg/day reduced cognitive dysfunction in AD rats through a dose-dependent reversal of imbalance in Th17/Treg cytokines ratio induced by a Aβ1-42 injection, while up-regulating Foxp3 mRNA expression and decreasing RORγt expression (Zhang Y. et al., 2015). In a similar study, it was also shown that MT repaired cognitive deficits in AD transgenic mice by blocking the Aβ/RAGE signaling pathway (Cui et al., 2016). Ni et al. also reported that MT injection reduced ibotenic acid (IBO)-induced increases in IL-1β content in AD rats, thereby protecting mitochondrial structure, improving energy metabolism, delaying neuronal apoptosis, and suppressing AD (Ni et al., 2006). Li et al. (2018) reported that the administration of MT effectively alleviated the learning and memory impairment and neuroinflammation of the LPS-induced AD mice model, with a potential mechanism involved in the inhibition of NADPH oxidase subunits GP91phox and P47phox protein expression. Yang et al. reported that MT could maintain and even strengthen the cellular nutrition of Aβ42 monomers by inhibiting the aggregation of Aβ42 monomers and synergizing with Aβ42 monomers. In addition, MT promotes the dissociation of immature Aβ42 oligomers to protect the morphological integrity of human neuroblastoma cell lines (SH-SY5Y). The underlying mechanism may be that the presence of MT-like metabolites in the human brain negatively regulates the formation of toxic Aβ42 oligomers (Yang et al., 2020). These results confirm that MT can be considered an effective multi-target compound for the prevention and treatment of AD. Furthermore, MT exerted a protective effect against PD induced in rats with 1−methyl−4−phenyl−1,2,3,6−tetrahydropyridine (MPTP), through blockage of the MPTP-induced overexpression of Nrf2 (Meng et al., 2017).

In an animal model of multiple sclerosis (MS), MT treatment (200 mg/kg/day) effectively improved the clinical symptoms of experimental autoimmune encephalomyelitis (EAE). Zhang M. L. et al. (2017) reported that MT enhanced local production of neurotrophin 3 (NT3) in microglia, astrocytes, and oligodendrocyte precursor cells, thereby protecting neural cells from tissue damage caused by CNS inflammation. It was shown for the first time that MT treatment promoted the differentiation and myelination oligodendrocytes during EAE *via* the activation of the PI3K/Akt/mTOR signaling pathway (Liu S. Q. et al., 2017). By directly activating the cAMP/PKA signaling pathway in astrocytes, MT up-regulated the production of the brain-derived neurotrophic factor (BDNF) and effectively protected nerve axons from CNS inflammation-induced damage. Furthermore, it was found that MT reduced the expression of NogoA, its receptor complex NgR/p75NTR/LINGO-1, and its downstream RhoA/ROCK signaling pathway, thereby promoting neuro-regeneration in damaged CNS during EAE (Kan et al., 2015; Kan et al., 2016). Studies have demonstrated that MT increased LPS-stimulated BV-2 cell viability and prevented microglial activation by suppressing the expression of heat shock protein 60 (HSP60) and toll-like receptor 4 (TLR-4), indicating that MT can be used to treat neurodegenerative diseases involving microglial activation (Zhang R. et al., 2017). It was demonstrated that MT alleviated anxiety and depression symptoms in a mouse model of burn injury by inhibiting JNK-mediated apoptosis/inflammatory signaling, oxidative stress, and reversing the burn-induced down-regulation of BDNF/VEGF in the hippocampus (Khan et al., 2020). Furthermore, Wu et al. (2018) demonstrated that MT possessed antidepressant-like effect on mice by activating the PI3K/Akt/mTOR signaling pathway in the hippocampus.

Based on these studies, MT may be used as an effective neuroprotective drug for the treatment of AD, PD, and CNS inflammation, neurobehavioral disorders, and it prevents LPS-induced neuronal injury.

### Anti-Allergic Effect of MT

Asthma is an allergic lung inflammatory disease whose occurrence is related to the expression levels of IL, IgE, and eosinophil. The Th2 type cytokines, including interleukin IL-4, IL-5, and IL-13, are essential factors for the initiation and transmission of inflammatory and allergic reactions (Agrawal and Shao, 2010; Bosnjak et al., 2011). To assess the anti-allergic activity of matrine, the potential mechanism of its action was studied by using an *in vivo* OVA-sensitized mice model and *in vitro* human bronchial epithelial cells (BEAS-2B). The results demonstrated that matrine alleviated eosinophil infiltration, airway hyperresponsiveness (AHR), and airway inflammation by inhibiting Th2 cytokines or the IL-4/IL-13/STAT-6 pathway in asthmatic mice. In addition, MT pretreatment significantly reduced the production of pro-inflammatory cytokines and eosinophil chemokines in activated beas-2b cells, and inhibited the expression of ICAM-1, thereby suppressing the adhesion of eosinophil to inflammatory BEAS-2B cells (Fu et al., 2014; Huang et al., 2014). These findings suggest that MT could be developed into a drug for the treatment of allergic asthma.

## Toxicology

Through *in vivo* experiments, the toxicity and target organs of Radix *Sophorae tonkinensis* extracts in ICR mice were studied, and the toxicities of its major bioactive components were compared. In these studies, administration of MT at doses of 118 and 154 mg/kg/day for 21 days produced major toxicity in the liver (Wang L. et al., 2017). In human normal liver HL-7702 cells, MT at doses of 2.5-5 mg/mL for 24 h, increased the contents of ALT, AST, ALP, LDH, and MDA, promoted the induction of cell apoptosis, and decreased the level of GSH (Zhang Q. et al., 2011). Furthermore, another study showed that MT at doses of 0.4 and 0.63 mM for 96 h induced a significant increase in zebrafish hepatocyte apoptosis, and down-regulated the oxidative stress-related gene zgc: 136383 and anti-apoptotic gene *EIF4BP3* (Guo S. et al., 2018). Previous studies confirmed that MT at doses of 1-4 mg/mL for 48h inhibited cell viability and induced cell cycle arrest and apoptosis in HepaRG and HL-7702 cells, most probably through a mechanism involving inhibition of the Nrf2 pathway and activation of the ROS-mediated mitochondrial apoptotic pathway (You et al., 2018; You et al., 2019).

MT has been reported to exert neurotoxic effects. Wang et al. reported that the half-lethal dose (LD_50_) of MT administered by intraperitoneal injection in Kunming mice was 157.13 mg/kg. In addition, histopathological observation showed that small softening foci were formed in the brain tissue of mice, and part of the nerve nuclei were necrotic or even broken, indicating that one of the main toxic target organs of MT is the nervous system (Wang et al., 2010). A study showed that when given at doses of 10 and 40 mg/kg/day for 60 days, MT inhibited the central nervous system of ICR mice, and impaired their balance and coordination (Liang et al., 2015). Investigation of the relationship between MT-induced neurotoxicity and oxidative stress in PC12 cells indicated that MT induced high level of oxidative stress after treatment with graded concentrations of MT (2-8 mM) for 48 h, resulting in a reduction of SOD and an increase in ROS and MDA, as well as activation of the mitochondria-dependent apoptotic pathway (Shen et al., 2017). In terms of reproductive toxicity, Luo et al. (2015) was the first to show that MT (100-200 µM) suppressed mouse sperm function by reducing sperm [Ca^2+^]_i_ and inhibiting the phosphorylation of ERK1/2. Moreover, further studies indicated that MT (10 ang50 mg/kg/day for 30 days) inhibited mouse sperm function through the [Ca^2+^]_i_-related mechanism *via* the CatSper channel, which is the main channel for controlling extracellular Ca^2+^ influx in mouse sperm (Luo et al., 2015; Luo et al., 2016). Moreover, it was found that the vinegar-processing method reduces the oral toxicity of *Sophora* alopecuroides L. containing MT and other MT-type alkaloids mainly due to a sharp decrease of cytisine (Zhang C. X. et al., 2018). These results indicated that the toxicity of *Sophora* alopecuroides L. might be related to dose and/or drug-drug interactions. In summary, the toxic effects of MT in clinical use remains unclear. These findings highlight the need to assess the risk of human exposure to MT.

## Pharmacokinetics

The absorption, distribution, metabolism, and excretion (ADME) processes of drugs *in vivo* are regulated by a variety of factors (dose, administration, and drug interaction). Wang and Huang (1992) was the first to report that intravenous administration of MT to rabbits at a dose of 40 mg/kg resulted in plasma concentration-time profiles consistent with the two-compartment open model, and the relationship between its effect and the effect of compartment concentration was consistent with the sigmoid E_max_ model (Wang and Huang, 1992). After oral administration of MT at doses of 300 mg/kg in beagle dogs, the drug was rapidly distributed and was eliminated from plasma with a terminal half-life (t_1/2β_) of 4.45 ± 0.83 h (Li Z. W. et al., 2010). Moreover, Zhao et al. (2010a) studied the pharmacokinetics of intramuscularly administered MT (30 mg/kg) in rats. The results showed that intramuscular administration of MT resulted in better absorption than oral administration, and the distribution from the central compartment to the peripheral compartment was faster. Moreover, its absolute bioavailability was higher than that of oral administration, and its t_1/2β_ and areas under concentration time curve (AUC) were 3.509 h and 90.984 mg/L h, respectively. It has been speculated that the pharmacological action was stronger with intramuscular administration than oral administration, and the maintenance time was also longer (Zhao et al., 2010a). It has also been shown that after an intravenous bolus injection of MT (2 mg/kg) to rats, the blood concentration of MT reached a maximum of 2412 ± 362 ng/ml, and subsequently quickly decreased. When the same dose of MT was orally administered to rats, it was easily absorbed and reached C_max_ of 94.6 ± 38.6 ng/ml after approximately 105 min (Yang et al., 2010). These results show that the C_max_ and T_max_ values of MT after oral administration were not significantly different from those reported by Wu et al., 2003; Zhang et al., 2008. Zhang et al. showed that the pharmacokinetics of MT in rats after oral administration of *flave Sophora scens* Ait. extracts (0.56 g/kg) was consistent with the two-compartment model, with C_max_ and T_max_ of 2529 ng/ml and 2.08 h, respectively (Zhang et al., 2010). In addition, Wu et al. performed pharmacokinetic studies on rats after oral administration of MT at a dose of 40 mg/kg, and reported C_max_ and T_max_ of approximately 3900 ng/ml and 50 min, respectively (Wu et al., 2003). In the Caco-2 cell model, the absorption permeability of MT was regulated by pH, while in the rat intestinal perfusion model, the absorption of MT was significantly different in the four intestinal segments (ileum, colon, duodenum, and jejunum) of the rat. In addition, Zhao et al. used *in vitro* rat liver microsomal model studies to reveal that MT could not be metabolized by CYP450 and UGT enzymes, indicating that MT may not undergo extensive first-pass metabolism after oral administration (Zhao et al., 2010a).

It has been suggested that the tissue distribution characteristics of MT solution (MS), MT liposome (ML), and MT stealth liposome (LML) were significantly different after intravenous administration of MT in rats at a single dose of 15 mg/kg. After administration of MT, it can be rapidly distributed to various tissues and organs, such as liver, spleen, and kidney. However, compared with the MS group, the LML group significantly reduced the uptake of MT by the liver and spleen, and improved the bioavailability of MT in rats (Wu et al., 2009). Tang et al. used an ultra-performance liquid chromatography–tandem mass spectrometry method to systematically study the pharmacokinetic behaviors of radix *Sophorae tonkinensis* extracts. The results indicated that oral administration of radix *S. tonkinensis* extracts exhibited different pharmacokinetic behavioral changes, when compared with pure oxymatrine (OMT). It was speculated that the potential mechanism was that other complex components in the extracts affected the transformation of OMT to MT (Tang et al., 2013). Moreover, after oral administration of *kushen-gancao* decoction, the concentrations of MT in the serum of rats was much lower than that of glycyrrhetinic acid (GA), suggesting that the binding of MT to organs was higher, while the absorption and blood distribution were lower (Wang et al., 2014).

## Drug-Drug Interactions of MT

Recently, enhancement of efficacy and/or toxicity due to drug-drug interactions has been a key consideration in the development of new drugs in pre-clinical and clinical investigations (Pai et al., 2018). Pu et al. showed that MT combined with cisplatin, 5-fluorouracil, and paclitaxel, respectively, effectively inhibited the proliferation of A549 cells and showed dose-dependent anticancer effects (Pu et al., 2018). It has been reported that a combination of MT and cisplatin synergistically inhibited proliferation, and induced apoptosis of rhabdomyosarcoma (RMS) RD cells, through a mechanism involving inhibition of mRNA expression of XIAP mRNA (Li et al., 2016). Another study found that MT and cisplatin synergistically suppressed the growth of urothelial bladder cancer cells (EJ, T24, BIU and 5637 cells) by inhibiting the VEGF/PI3K/Akt signaling pathway (Liao et al., 2017). Moreover, the protein expression levels of topoisomerase (TOPO) I, Bax, and caspase-3 were up-regulated in HT29 cells treated with a combination of MT and irinotecan (Duan et al., 2017). Rong et al. reported that MT combined with platinum-based doublet chemotherapy (PBDC) had a lower adverse reaction rate, higher response rate (RR), disease control rate (DCR) and mean survival time (MST), and better quality of life (QOL) than PBDC alone (Rong et al., 2015). It has been reported that the co-administration of MT and oxymatrine synergistically inhibits ATP production and cell proliferation of human umbilical vein endothelial cells (ECV304), thereby achieving indirect antitumor effects against tumor angiogenesis (Wang S. J. et al., 2017). All of these studies showed that MT could strengthen the efficacy of many anticancer drugs through drug-drug interactions.

In a rat hepatic stellate cell line (HSC-T6), it was reported that a combination of MT and glycyrrhizin (100 µM MT+glycyrrhizin) inhibited proliferation of activated HSCs and reduced levels of collagen I and hexadecenoic acid (HA). It was also found that combination of MT and glycyrrhizin, 1 mg/mL MT and 1 mg/mL glycyrrhizin, given at a dose of 0.1 mL/100g body weight significantly reduced serum laminin (LN), HA and procollagen type-III (PC-III) levels in a rat model of CCl_4_-induced liver fibrosis, when compared with MT or glycyrrhizin alone (Zhao et al., 2012). Administration of a mixture of 1 mg/mL glycyrrhizin and 1 mg/mL MT to mice at a dose of 0.5 mL/20g body weight decreased mortality of acetaminophen-overdosed mice, reduced acetaminophen-induced hepatotoxicity, and decreased the area and number of glutamyl transpeptidase (-GT)+positive foci, thereby restoring liver function and preventing liver cancer (Wan et al., 2009). Furthermore, *in vitro* experiments showed that a combination of MT with lamivudine dose-dependently inhibited HBV-DNA replication and secretions of hepatitis Be antigen (HBeAg) and hepatitis B surface antigen (HBsAg) in HepG2 2.2.15 cell (Ma et al., 2013). Another study showed that combination of sub-MIC MT and erythromycin had a synergistic inhibitory effect on plankton and adhesion of *Staphylococcus epidermidis* (Guan et al., 2013). It has been reported that a combination of MT and ciprofloxacin significantly decreased the expression level of AcrA, resulting in the inhibition of multidrug-resistant phenotypes in Gram-negative bacteria (Pourahmad and Mohammadi, 2018). In a non-tumorigenic human skin keratinocytes (HaCaT) model, MT combined with acitretin negatively regulates the phosphorylation of PI3K/Akt/mTOR signaling pathway, and plays a synergistic role in inducing autophagy and cell cycle G0/G1 phase arrest (Jiang W. W. et al., 2019). Studies have shown that the combination of MT and lycopene possessed a synergistic protective effect on a LPS-induced acute lung injury (ALI) mouse model through the inhibition of the NF-κB pathway (Li W.W. et al., 2019). Rong et al. reported that MT combined with other drugs (such as Cisplatin, interleukin-11, and bleomycin) has a synergistic effect, which can effectively improve the control of malignant pleural effusion and reduce the incidence of adverse reactions (Rong et al., 2015). Taken together, these results indicated that MT combined therapy showed excellent efficacy.

Qi et al. (2012) studied the effect of glycyrrhizin on MT pharmacokinetics in rats. Compared with the MT alone administration group, the C_max_ of MT in the MT and glycyrrhizin combination group decreased from 6.861 ± 0.635 mg/L to 4.122 ± 0.965 mg/L, and the AUC of MT from 47.105 ± 7.062 mg/L h decreased to 35.508 ± 5.024 mg/L h (Qi et al., 2012). Another similar study also suggested that the C_max_ and AUC of MT reduced by 32.8% and 34.9% after the combination of Sophorae flavescentis radix (Kushen) and Glycyrrhizae radix et rhizoma (Gancao) (Shi et al., 2015). Furthermore, Zhao et al. (2010a) reported that the AUC and C_max_ of MT were significantly decreased when MT was administered in combination with ceftiofur hydrochloride (Zhao et al., 2010b). These results indicated that the combination of MT and other drugs (such as glycyrrhizin and ceftiofur hydrochloride) results in a serious decrease in the plasma concentration and bioavailability of MT, which may adversely affect the pharmacological effects of MT. Therefore, in the clinical combination, we should fully evaluate the pharmacological effects, toxicity, and pharmacokinetic characteristics of MT to obtain better efficacy and drug safety.

## Conclusions and Future Perspectives

**Figure 2:**
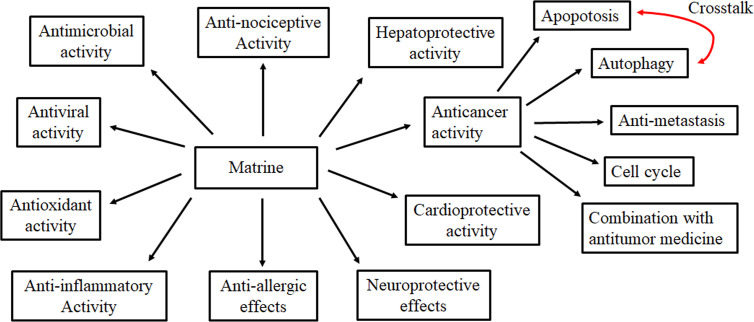
Pharmacological activities of Matrine.

MT is a quinolizidine alkaloid with potential pharmacological benefits, including anti-cancer, anti-inflammatory, anti-oxidant, antiviral, antimicrobial, anti-fibrosis, anti-allergic, antinociceptive, hepatoprotective, cardioprotective, and neuroprotective effects (**Figure 2**). Previous studies have shown that MT may be one of the valuable options for the prevention and therapy of cancers, hepatitis, skin diseases, allergic asthma, diabetic cardiomyopathy, analgesic, AD, PD, and CNS inflammation. In addition, MT exerts significant anti-cancer effects, through a mechanism involving the regulation of gene and protein expressions of pathways involved in apoptosis, autophagy, cell invasion, and metastasis, as well as cell cycle arrest. Furthermore, studies have suggested that MT should be used in combination with a variety of chemotherapy drugs and alternative therapies to treat cancer more effectively. Therefore, in the clinical combination of MT, it is necessary to assess the effect of MT on the metabolism of other drugs.

Detailed studies of MT with respect to the underlying mechanisms of its effects have shown that its anti-tumor effects are associated with the inhibition of various proteins and genes that are overexpressed in cancer such as IL-6, JAK2, STAT3, PCNA, VEGF-A, NF-κB, MMP-9/MMP-2, phosphorylated AKT, p-65, VEGFR1, epidermal growth factor (EGF), VASP, and SQSTM1/p62. However, the expression levels of Bax, Fas, XIAP, mitochondrial membrane potential, p-ERK, CIAP, p53, p21, p27, P-gp, MRP, caspase-3, caspase-9, and LC3-II are up-regulated. The effect is primarily mediated by signaling pathways involving JAK2/STAT3, AKT, miR-21/PTEN/AKT, Akt/mTOR, and NF-κB. Consistently, these data have strongly established that MT has the capacity to influence various events involved in cell apoptosis, autophagy, cell metastasis, invasion, and cell cycle arrest. These data clearly support its traditional use in cancer therapy. In this paper, a wide range of pharmacological effects and molecular mechanisms of MT have been systematically summarized to further support the viewpoint that MT possesses a broad application prospect (**Table 1**).

**Figure 3:**
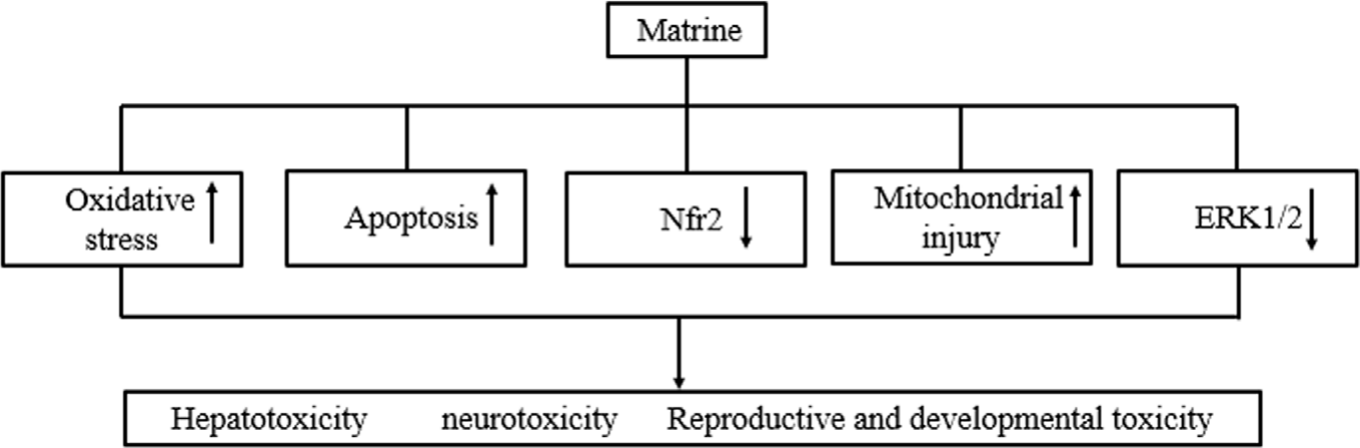
Toxicity and mechanism of Matrine.

Unfortunately, MT has been reported to have serious side effects, including hepatotoxicity, neurotoxicity, and reproductive and developmental toxicity, thereby limiting its clinical use (**Table 2**). The potential toxic mechanisms involve the activation of receptor-mediated and mitochondrial-dependent apoptotic pathways, as well as the inhibition of the Nrf2 and ERK pathways (**Figure 3**). It is worth noting that MT salvianolic acid B salt treatment at doses of 25, 50, or 100 mg/kg/day for 56 days ameliorated fibrosis induced by carbon tetrachloride injection, but without significant hepatotoxicity (Gao et al., 2012). The mechanism involved might be that MT and salvianolic acid B combined to form a salt which enhanced the efficacy of the treatment and reduced the risk of toxicity. Further studies are needed on the mechanism involved. In addition, as summarized in **Tables 1** and **2**, the toxic doses of MT significantly affected the mitochondrial apoptotic pathway, Nrf2 pathway and ERK1/2 pathway. These findings indicate that MT-induced toxicity has an underlying mechanism similar to that of the pharmacological effects of MT. Therefore, further studies are needed for a closer investigation of the relationship between the efficacy and toxicology of MT. Moreover, the appropriate clinical dose range and exposure mechanism for different dosing times and patient disease conditions need to be determined. Taken together, while pursuing greater efficacy, attention should be paid to reducing the toxicity of MT. Future studies should focus on the combination with other drugs, determination of a single dose range, structural optimization, formulation, and establishment of a toxicity warning system.

**Table 2:** Toxicity of matrine.

Cell lines/model	Dose	Activity/mechanism(s) of action	Application	Reference
Zebrafish	0.4 and 0.63 mM, 96 h	Regulates oxidative stress and apoptosis-related gene	*In vivo*	Guo P. et al. (2018)
ICR and C57BL/6 mice	10 and 40 mg/kg/day for 60 days	Increases activity of AST and ALT	*In vivo*	Liang et al. (2015)
HL-7702 cells	1-4 mg/mL, 48h	Inhibits the activation of Nrf2 pathway	*In vitro*	You et al. (2019)
HepaRG cells	1-4 mg/mL, 48h	Induces ROS-mediated mitochondrial apoptotic pathway	*In vitro*	You et al. (2018)
PC12 cells	2-8 mM, 48 h	Activation of the mitochondria-dependent apoptotic pathway	*In vitro*	Shen et al. (2017)
C57BL/6J mice	10 ang 50 mg/kg/day for 30 days	Reduces sperm [Ca^2+^]i by CatSper channel, suppresses the phosphorylation of ERK1/2.	*In vivo*	(Luo et al., 2015; Luo et al. 2016)

In this study, we summarize and discuss the factors affecting MT ADME based on the latest understanding of MT pharmacokinetics (**Table 3**). At present, oral and injection routes are widely used in clinical applications of MT. The oral administration is relatively simple and convenient, and can meet the treatment of some specific diseases. After intravenous administration of MT, it is quickly absorbed, and it reaches the site of action through blood circulation, with high bioavailability, no first-pass effect, and accurate dose. However, in future drug development and application, attention should be paid to the emergence of other toxicity problems caused by increase in the content of MT.

**Table 3:** Pharmacokinetics of matrine.

Model	Route of administration	Drug/Dose	Characteristics of pharmacokinetics	Reference
Rabbit	Intravenous	MT, 40 mg/kg	Two-compartment open model	Wang and Huang, 1992
Beagle dog	Oral	MT, 300 mg/kg	t_1/2β_ of 4.45 ± 0.83 h	Li Z. W. et al., 2010
Rat	Intramuscular	MT, 30 mg/kg	t_1/2β_ and AUC were 3.509 h and 90.984 mg/L h, respectively.	Zhao et al. (2010b)
Rat	Intravenous bolus	MT, 2 mg/kg	C_max_ of 2412 ± 362 ng/ml	Yang et al., 2010
Rat	Oral	MT, 2 mg/kg	C_max_ of 94.6 ± 38.6 ng/ml	Yang et al., 2010
Rat	Oral	*flave Sophora scens* Ait. extracts, 0.56 g/kg	Two-compartment model, with C_max_ and T_max_ of 2529 ng/ml and 2.08 h, respectively	Zhang et al., 2008
Rat	Oral	MT, 40 mg/kg	C_max_ and T_max_ of approximately 3900 ng/ml and 50 min, respectively	Wu et al., 2003
Rat	Intravenous	MT, 15 mg/kg	Rapidly distributed to various tissues and organs, such as liver, spleen and kidney.	Wu et al., 2009
Rat	Oral	radix *S. tonkinensis* extracts (equivalent to the administration of 2 mg/kg OMT)	Other complex components in the extract may influence the transformation from OMT to MT	Tang et al., 2013

This paper reviewed the progress of research on MT in recent years, highlighting its preponderant pharmacological activity and inevitable side effects. MT possesses a variety of pharmacological effects, including antioxidant, anti-inflammatory, antiviral, antinociceptive, neuroprotective, and cardioprotective properties. Although the pharmacological mechanism of MT has been widely investigated, the mechanism of its anti-tumor action and its synergistic treatment with other drugs needs to be further studied. In general, future drug development of MT needs to focus on ameliorating its oral bioavailability, reducing toxicity, and improving efficacy.

## Author Contributions

LY and CY wrote the manuscript. YD, WW, MS, JL, and BM analyzed the data, while LP, YZ, and ZZ made the pictures and tables. XD, XY, and JN designed the research and revised the primary manuscript. All authors contributed to the article and approved the submitted version.

## Funding

This work was financially supported by the National Natural Science Foundation of China (81703715).

## Conflict of Interest

The authors declare that the research was conducted in the absence of any commercial or financial relationships that could be construed as a potential conflict of interest.
